# Publisher Correction: Serotonin signals through a gut-liver axis to regulate hepatic steatosis

**DOI:** 10.1038/s41467-018-08085-x

**Published:** 2019-01-08

**Authors:** Wonsuk Choi, Jun Namkung, Inseon Hwang, Hyeongseok Kim, Ajin Lim, Hye Jung Park, Hye Won Lee, Kwang-Hyub Han, Seongyeol Park, Ji-Seon Jeong, Geul Bang, Young Hwan Kim, Vijay K. Yadav, Gerard Karsenty, Young Seok Ju, Chan Choi, Jae Myoung Suh, Jun Yong Park, Sangkyu Park, Hail Kim

**Affiliations:** 1 Graduate School of Medical Science and Engineering, KAIST, Daejeon, 34141 Republic of Korea; 2 Department of Biochemistry, Yonsei University Wonju College of Medicine, Wonju, 26426 Republic of Korea; 3 Biomedical Science and Engineering Interdisciplinary Program, KAIST, Daejeon, 34141 Republic of Korea; 4 Department of Internal Medicine, Yonsei University College of Medicine, Yonsei Liver Center, Severance Hospital, Seoul, 03722 Republic of Korea; 5 Center for Bioanalysis, Division of Metrology for Quality of Life, Korea Research Institute of Standards and Science, Daejeon, 34113 Republic of Korea; 6 Biomedical Omics Group, Korea Basic Science Institute, Chungbuk, 28119 Republic of Korea; 7 Department of Genetics and Development, Columbia University Medical Center, New York, NY 10032 USA; 8 Department of Pathology, Chonnam National University Medical School, Gwangju, 61469 Republic of Korea; 9 Department of Biochemistry, College of Medicine, Catholic Kwandong University, Gangneung, 25601 Republic of Korea; 10 KAIST Institute for the BioCentury, KAIST, Daejeon, 34141 Republic of Korea

Correction to: *Nature Communications*; 10.1038/s41467-018-07287-7, published online 16 November 2018

The originally published version of this Article contained an error in Fig. 2. In panel g, the image of brown adipose tissue from SCD-fed *Tph1* GKO mice (top-right) was inadvertently replaced with the equivalent image of SCD-fed WT mice (top-left) during assembly of the figure. This error has now been corrected in both the PDF and HTML versions of the Article.

