# Supplementary material for: Serotonin signals through a gut-liver axis to regulate hepatic steatosis
Source: Nat Commun. 2018 Nov 16;9:4824. doi: 10.1038/s41467-018-07287-7 (PMC6240035; doi:10.1038/s41467-018-07287-7)
Supplement: Supplementary file 1 — Supplementary Information [file 41467_2018_7287_MOESM1_ESM.pdf]

Supplementary Information

Serotonin signals through a gut-liver axis to regulate hepatic steatosis

Choi et al.

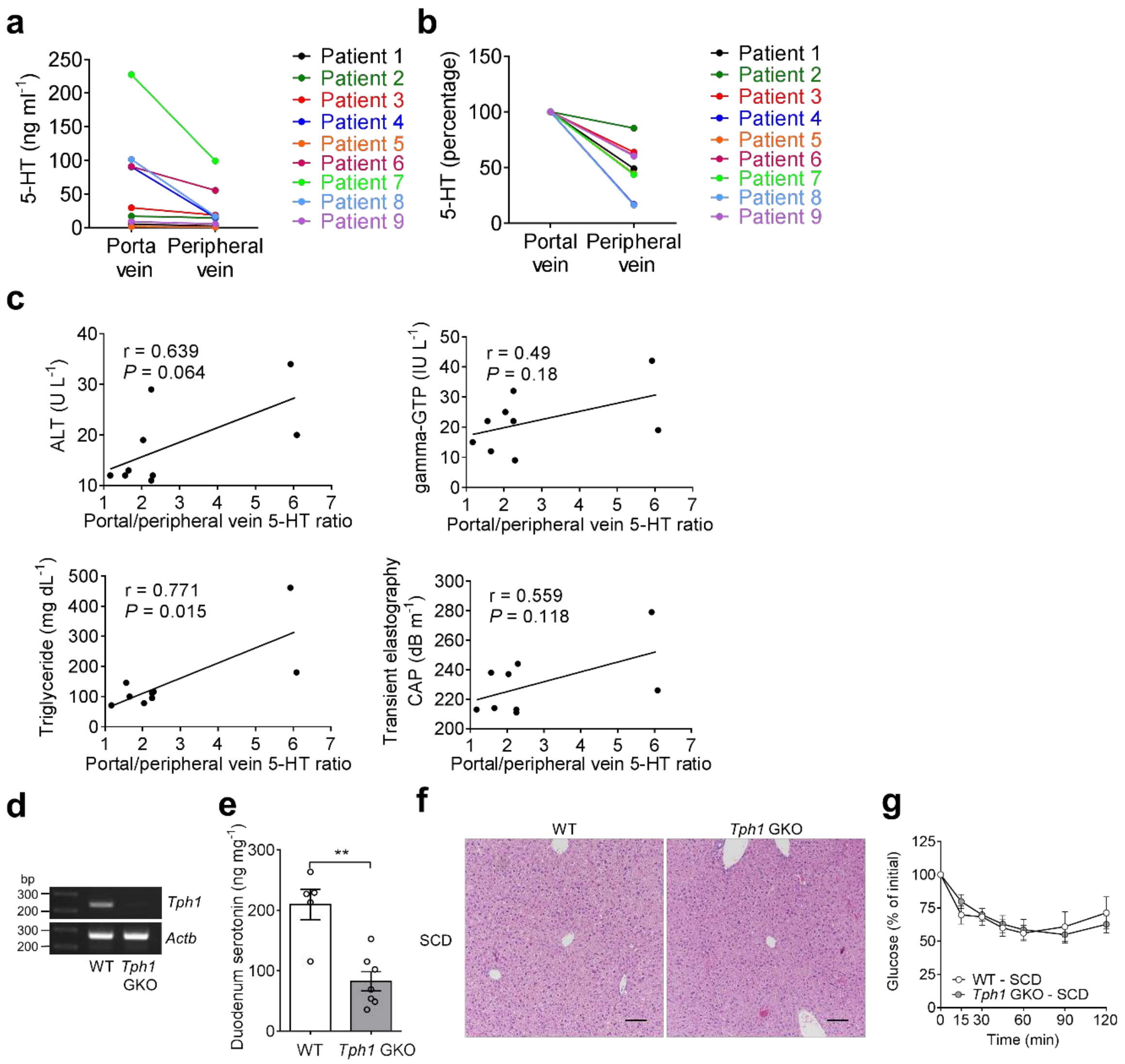

Supplementary Figure 1. Inhibiting gut-derived 5-HT does not affect hepatic lipid metabolism in SCD-fed mice. (**a**) Plasma 5-HT concentrations of portal blood and peripheral blood in humans. n = 9 per group. (**b**) Plasma 5-HT concentrations of portal blood and peripheral blood in humans. Portal blood concentration set as 100%. n = 9 per group. (**c**) Scatter plots of values for portal/peripheral blood 5-HT ratio versus ALT, gamma-GTP, TG and transient elastography CAP in humans. n = 9 per group. These data were compared with one another by calculation of the Pearson's *r* correlation coefficient. (**d**) Representative RT-PCR data of *Tph1* gene expression in duodenum from 20 week old WT and *Tph1* GKO mice. (**e**) Duodenal 5-HT levels in WT and *Tph1* GKO mice. n = 5-7 per group. (**f**) Representative liver histology by H&E staining from SCD fed WT and *Tph1* GKO mice. (**g**) Intraperitoneal insulin tolerance test (IPITT) after 4 h fasting of SCD fed WT and *Tph1* GKO mice. n = 5 per group. Scale bars, 100µm. Data are expressed as the means ± standard error of the mean (SEM). **P*<0.5, ***P*<0.01, ****P*<0.001, Student's *t*-test (e, g).

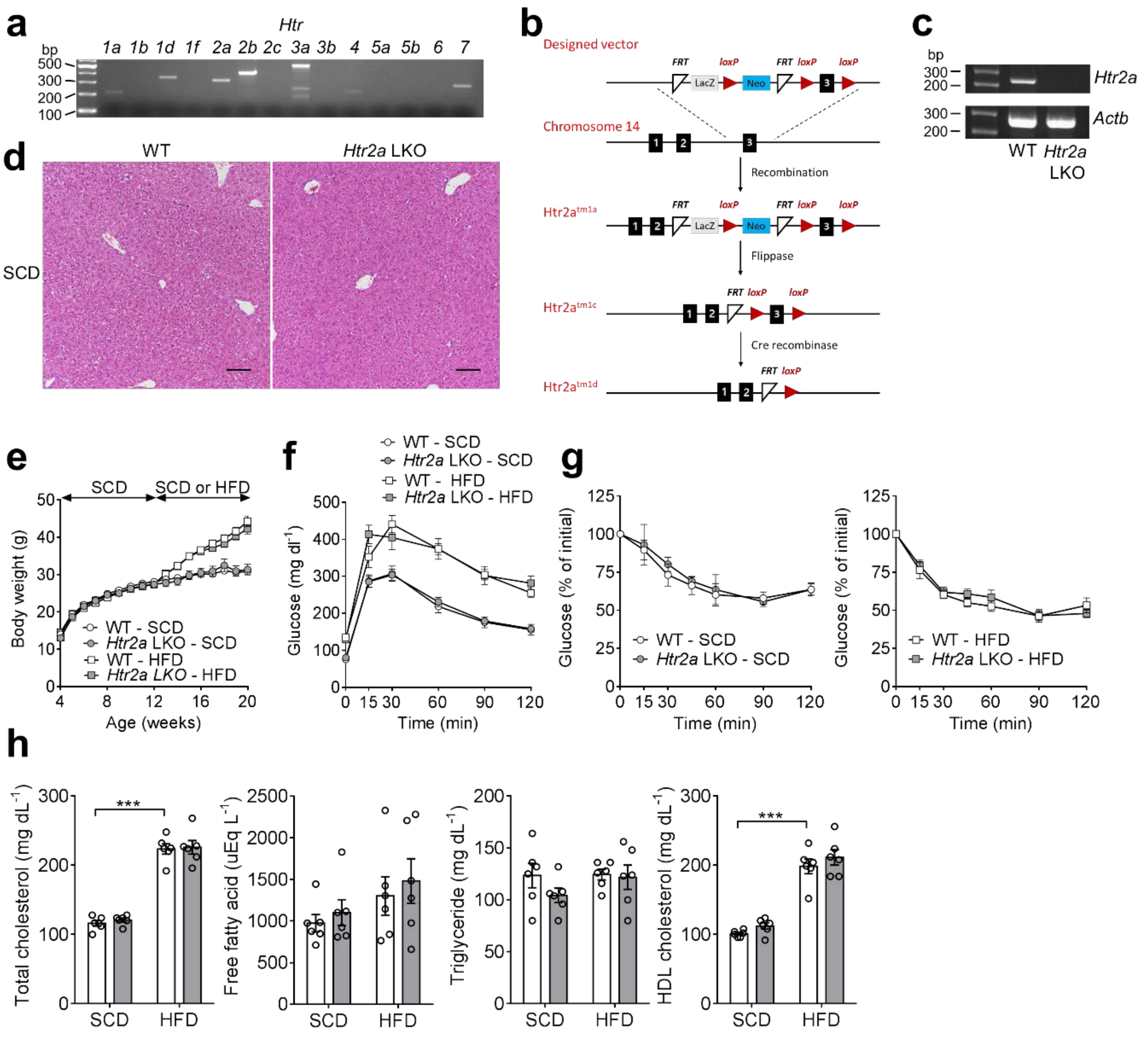

Supplementary Figure 2. Liver-specific deletion of *Htr2a* does not affect systemic energy metabolism. (**a**) mRNA expression of *Htr*s as assessed by RT-PCR in liver from 8 week old C57BL/6J mice. (**b**) Schematic of gene targeting strategy to generate the *Htr2a* floxed allele. (**c**) Representative RT-PCR data of *Htr2a* gene expression in liver from 20 week old WT and *Htr2a* LKO mice. (**d-h**) 12 week old WT and *Htr2a* LKO mice were fed SCD or HFD for 8 weeks. (**d**) Representative liver histology by H&E staining from SCD fed WT and *Htr2a* LKO mice. Scale bars, 100µm. (**e**) Body weight trend. n = 6-10 per group. (**f**) Intraperitoneal glucose tolerance test (IPGTT) after 16 h fasting. n = 6 per group. (**g**) Intraperitoneal insulin tolerance test (IPITT) after 4 h fasting. n = 6 per group. (**h**) Plasma total cholesterol, free fatty acid, triglyceride, and HDL cholesterol levels. n = 6 per group. Data are expressed as the means ± standard error of the mean (SEM). **P*<0.5, ***P*<0.01, ****P*<0.001, Student's *t*-test (**g**) or one-way ANOVA with *posthoc* Tukey's test (**e,f,h**).

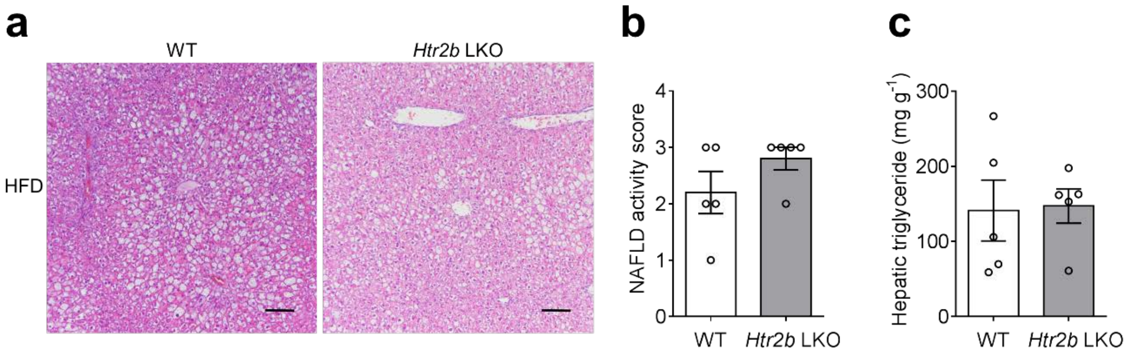

Supplementary Figure 3. HTR2B does not mediate the lipogenic action of GDS in the liver. (**a-c**) 12 week old mice were fed HFD for 8 weeks. (**a**) Representative liver histology by H&E staining from HFD fed WT and *Htr2b* LKO mice. Scale bars, 100μm. (**b**) NAFLD activity scores of HFD fed WT and *Htr2b* LKO mice. n = 5 per group. (**c**) Hepatic triglyceride levels. n = 5 per group.

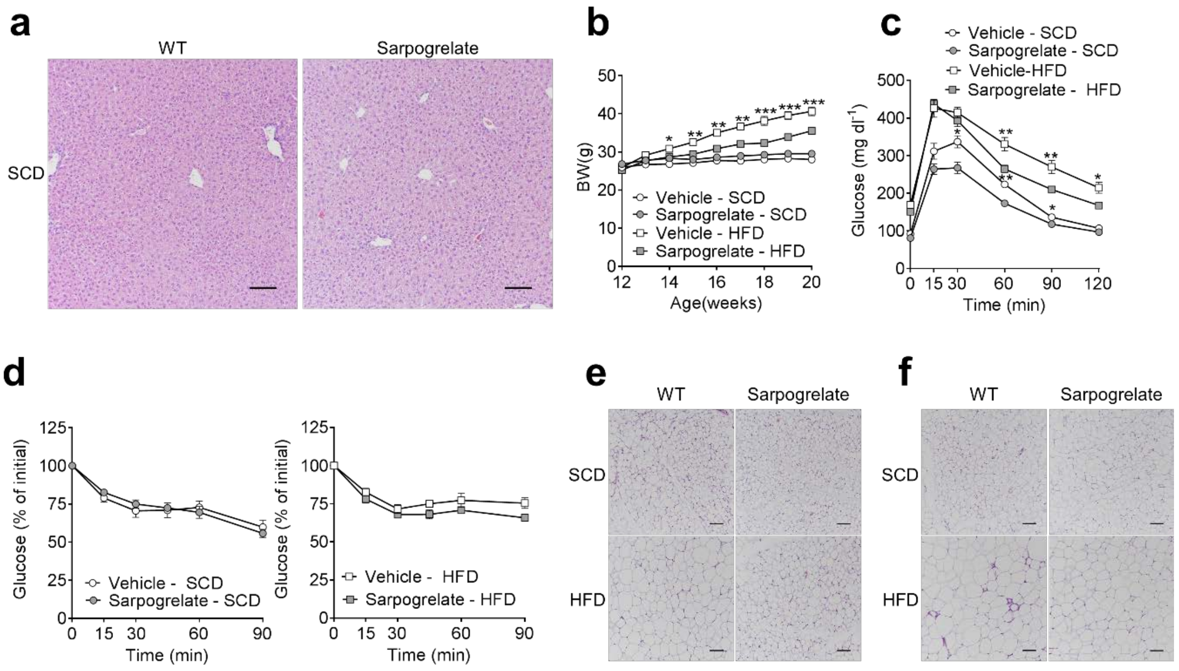

Supplementary Figure 4. HTR2A antagonist ameliorates HFD-induced obesity. (**a-f**) 12 week old mice were fed SCD or HFD for 8 weeks and were treated with vehicle or sarpogrelate daily per os. (**a**) Representative liver histology by H&E staining from SCD fed vehicle and sarpogrelate treated mice. Scale bars, 100μm. (**b**) Body weight trend. n = 5-6 per group. (**c**) Intraperitoneal glucose tolerance test (IPGTT) after 16 h fasting. n = 5-6 per group. (**d**) Intraperitoneal insulin tolerance test (IPITT) after 4 h fasting. n = 5-6 per group. (**e, f**) Representative iWAT (**e**) and eWAT (**f**) histology by H&E staining. Scale bars, 100μm. Data are expressed as the means ± standard error of the mean (SEM). **P*<0.5, ***P*<0.01, ****P*<0.001, Student's *t*-test (**d**) or one-way ANOVA with *posthoc* Tukey's test (**b,c**).

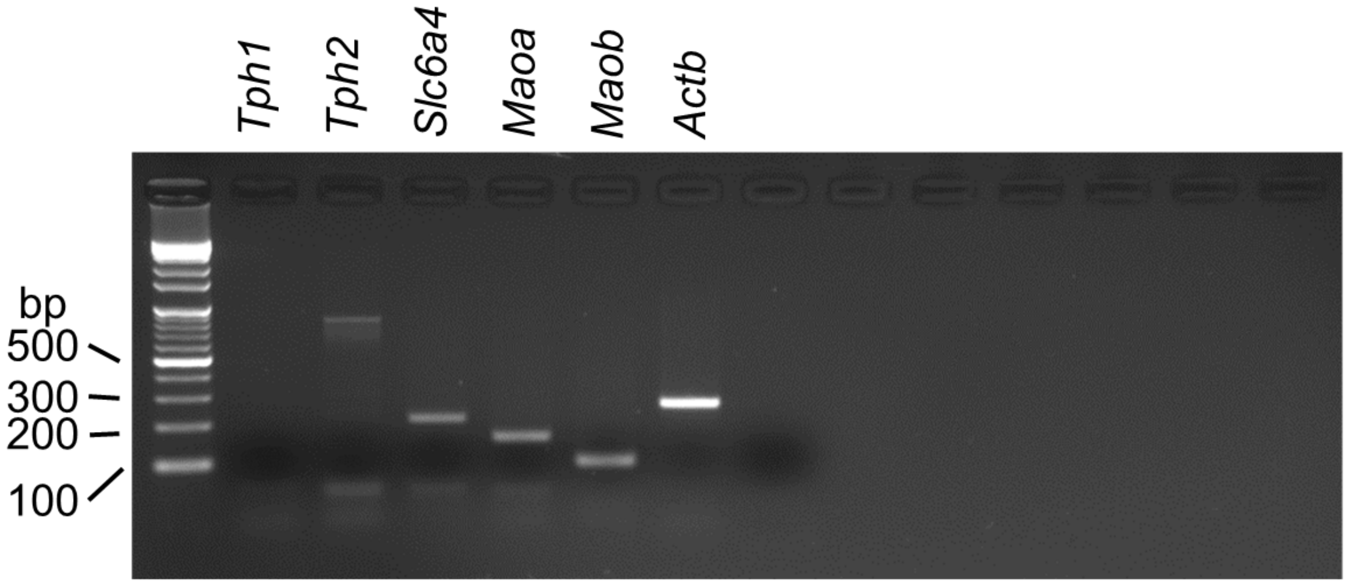

Supplementary Figure 5. Uncropped gel photo shown in Figure 1.

Supplementary Table 1. List of primers used in this study.

Gene name	Forward primer (5' to 3')	Reverse primer (5' to 3')
Acaca	CAGTAACCTGGTGAAGCTGGA	GCCAGACATGCTGGATCTCAT
Acat1	CCCCATTGATTTTCCACTTG	AGCACAACCACACTGAATGC
Acox1	CACGGCTATTCTCACAGCAG	CAGGCTGTTAATGTCCACCA
Actb	GGTACCACCATGTACCCAGG	GAAAGGGTGTAAAACGCAGC
Agpat1	GCGCAATGTCGAGAACATGA	TCATTCCAAGCAGGTCGAGG
Apob	TACTTCCACCCACAGTCCCCT	CCTTAGAAGCCTTGGGCACAT
Cd36	TGGCCAAGCTATTGCGACAT	ACACAGCGTAGATAGACCTGC
Col1a1	ACATGTTCAGCTTTGTGGACC	TAGGCCATTGTGTATGCAGC
Col3a1	GGAACCTGGTTTCTTCTCACC	TAGGACTGACCAAGGTGGCT
Col4a1	GTCTGGCTTCTGCTGCTCTT	CACATTTTCCACAGCCAGAG
Cpt1a	AGCTCGCACATTACAAGGACA	CCAGCACAAAGTTGCAGGAC
Fabp1	TGAAGGCAATAGGTCTGCCC	GTCATGGTCTCCAGTTCGCA
Fabp2	CTTCGGGAACCACAGGTCTTC	CATAGCAAGGCCTGTCCCATAC
Fasn	AAGCGGTCTGGAAAGCTGAA	AGGCTGGGTTGATACCTCCA
Fatp5	TTCGAAAGAACCAACCCTTCCT	GCGTCGTACATTCGCAACAA
F4/80	TCAAGGACACGAGGT TGCTGA	CCAAGGGGCCAATCTGGAA
Gpat1	CCACAGAGCTGGGAAAGGTT	GTGCCTTGTGTGCGTTTCAT
Htr1a	TCAGCTACCAAGTGATCACCTCT	GTCCACTTGTTGAGCACCTG
Htr1b	TGCTCCTCATCGCCCTCTATG	CTAGCGGCCATGAGTTTCTTCTT
Htr1d	CCTCCAACAGATCCCTGAATG	CAGAGCAATGACACAGAGATGCA
Htr1f	TGTGAGAGAGAGCTGGATTATGG	TAGTTCCTTGGTGCCTCCAGAA
Htr2a	AGCTGCAGAATGCCACCAACTAT	GGGATTGGCATGGATATACCTAC
Htr2b	AAATAAGCCACCTCAACGCCT	TCCCGAAATGTCTTATTGAAGAG
Htr2c	TTCTTAATGTCCCTAGCCATTGC	GCAATCTTCATGATGGCCTTAGT
Htr3a	AAATCAGGGCGAGTGGGAGCTG	GACACGATGATGAGGAAGACTG
Htr3b	CGTGTGGTACCGAGAGGTTT	GGATGGGCTTGTGGTTTCTA
Htr4	ATGGACAAACTTGATGCTAATGTGA	TCACCAGCACCGAAACCAGCA

Htr5a	GATTGACTTCAGTGGGCTCG	AAAGTCAGGACTAGCACTCG
Htr5b	GGAGCCTTCTACCTGCCTCT	ATGAGCTCCGTCAGGAAGAA
Htr6	CCTCACATGGCTGGGATACT	ATCTGAGTTGGGTGGCAGAG
Htr7	CTCGGTGTGCTTTGTCAAGA	TTGGCCATACATTTCCCATT
Maoa	GCGGTACAAGGGTCTGTTCC	CAGCCAATCCTGAGATGCCG
Maob	GGGCGGCATCTCAGGTATGG	AAGTCCTGCCTCCTACACGG
Mcad	GGTTTGGCTTTTGGACAATG	TGACGTGTCCAATCTACCACA
Mcp1	GGCTCAGCCAGATGCAGTTAA	AGCCTACTCATTGGGATCATCTT
Mogat1	TTGACCCATGGTGCCAGTTT	GTGGCAAGGCTACTCCCATT
Mttp	TCTCACAGTACCCGTTCTT	TCTTCTCCGAGAGACATATCC
Ppara	AGAAGTTGCAGGAGGGGATT	TCGGACTCGGTCTTCTTGAT
Pparg	GGTGTGATCTTAACTGCCGGA	GCCCAAACCTGATGGCATTG
Slc6a4	CGCAGTTCCCAGTACAAGC	GAAGGCCCCTCCACCATTCTG
Srebp1c	GGAGCCATGGATTGCACATT	GGCCCGGGAAGTCACTGT
Tgfb1	GGGCTACCATGCCAACTTCTG	GAGGGCAAGGACCTTGCTGTA
Timp1	CCCTGCTCAGCAAAGAGC	TCACTCTCCAGTTTGCAAGG
Tph1	ACCATGATTGAAGACAACAAGGAG	TCAACTGTTCTCGGCTGATG
Tph2	GCCATGCAGCCCGCAATGATGATG	CAACTGCTGTCTTGCTGCTC
Ucp1	CTTTGCCTCACTCAGGATTGG	ACTGCCACACCTCCAGTCATT

Supplementary Table 2. Clinical characteristics of study participants.

Patient	1	2	3	4	5	6	7	8	9
**Age**	21	24	37	48	58	37	42	36	53
**Sex**	Male	Male	Male	Male	Male	Male	Female	Male	Female
**Height (m)**	1.76	1.74	1.78	1.7	1.62	1.85	1.62	1.68	1.61
**Weight (kg)**	75	65	75	74	53	81	63	68	53.5
**BMI(kg m$^{2-1}$)**	24.21	21.47	23.67	25.61	20.2	23.67	24.01	24.1	20.64
**Hb (g dL^{-1})**	16.9	15.5	15.2	15.4	13.3	15.4	10.3	14.1	13.9
**WBC (10^{3} uL^{-1})**	5	5.02	7.37	8.46	6.57	5.72	7.47	5.7	5.69
**Seg (%)**	46.3	64.9	62.4	55.9	59.6	49.7	74.4	57.1	37.3
**Lymph (%)**	44.1	27.1	27.4	32.4	30.5	37.6	18.5	33.9	45
**PLT (10^{3} uL^{-1})**	200	255	219	270	239	281	327	276	253
**Ca (mg dL^{-1})**	9.6	9.7	8.9	9.3	9.5	9.8	9.3	9.4	9.7
**P (mg dL^{-1})**	3.8	3.6	3.3	2.9	4	3.5	4.4	3.7	3.7
**Glucose (mg dL^{-1})**	94	95	98	98	117	101	103	105	92
**BUN (mg dL^{-1})**	16.5	7.6	8.1	12.6	10.2	12.8	12.2	14.1	12.9
**Cr (mg dL^{-1})**	0.79	0.86	0.83	0.86	0.65	0.94	0.9	0.69	0.67
**Uric acid (mg dL^{-1})**	6	4.8	5.9	5.4	5	7.2	5.5	5.1	4.7
**Cholesterol (mg dL^{-1})**	133	154	139	212	211	281	209	180	219
**Total protein (g dL^{-1})**	7.2	7.3	6.9	6.8	6.4	7.7	7.3	7.4	7.1
**Albumin (g dL^{-1})**	4.7	4.9	4.5	4.4	4.5	4.6	4.6	4.7	4.6
**ALP (IU L^{-1})**	79	72	70	81	64	51	36	69	64
**AST (IU L^{-1})**	16	14	13	17	21	29	14	26	17
**ALT (IU L^{-1})**	19	12	12	34	11	29	12	20	13
**Bilirubin (mg dL^{-1})**	1.2	1	0.6	0.9	0.6	0.8	0.9	1.3	0.8
**gamma-GTP (IU L^{-1})**	25	15	22	42	22	32	9	19	12
**TG (mg dL^{-1})**	78	71	146	461	95	112	116	180	100
**HDL cholesterol (mg dL^{-1})**	44	62	36	37	43	68	51	50	75
**LDL cholesterol (mg dL^{-1})**	78	81	85	89	142	194	137	104	118
**Na (mmol L^{-1})**	142	142	143	140	142	141	140	143	142
**K (mmol L^{-1})**	4.4	4	4.9	4.6	4.5	4.7	4.2	4.5	4.7
**Cl (mmol L^{-1})**	102	100	105	102	102	100	101	103	101

**PT (Sec)**	11.5	12	11.2	10.7	10.6	10.5	11.6	10.5	10.4
**PT (%)**	98	92	100	100	100	100	91	100	100
**PTT (sec)**	37.3	32.7	33.4	29.3	29.8	29.6	32.7	31.2	31.2
**Portal vein serotonin (ng ml^{-1})**	5.3	17.1	29.6	90.6	1.8	91	227.6	101.6	9.1
**Systemic vein serotonin (ng ml^{-1})**	2.6	14.6	18.9	15.3	0.8	55.5	99.3	16.7	5.5
**Portal vein/systemic vein serotonin**	2.04	1.17	1.57	5.92	2.25	1.64	2.29	6.08	1.65
**Fibroscan LSM (kPa)**	5.3	4.9	3.1	4.6	4.9	3.6	4.6	4.5	8.5
**Fibroscan CAP (dB m^{-1})**	237	213	238	279	213	211	244	226	214

BMI, body mass index; Hb, hemoglobin; Seg, segmented neutrophil; Lymph, lymphocyte; PLT, platelet; BUN, blood urea nitrogen; ALP, alkaline phosphatase; AST, aspartate transferase; ALT, alanine transferase; TG, triacylglycerol; HDL, high-density lipoprotein; LDL, low-density lipoprotein; PT, prothrombin time; PTT, partial thromboplastin time; LSM, liver stiffness measure; CAP, controlled attenuation parameter.
