# Supplementary material for: Serotonin signals through a gut-liver axis to regulate hepatic steatosis
Source: Nat Commun. 2018 Nov 16;9:4824. doi: 10.1038/s41467-018-07287-7 (PMC6240035; doi:10.1038/s41467-018-07287-7)
Supplement: Supplementary file 3 — Description of Additional Supplementary Files [file 41467_2018_7287_MOESM3_ESM.pdf]

Description of Additional Supplementary Files

File Name: Supplementary Data 1

Description: List of GO gene sets significantly enriched or less enriched in livers of HFD-fed Htr2a LKO mice compared to WT littermates.

Sheet 1: GO gene sets significantly enriched in livers of HFD-fed Htr2a LKO mice . Lists of significantly enriched GO gene sets in livers of HFD-fed Htr2a LKO mice analyzed by GSEA.

Sheet 2: GO gene sets significantly less enriched in livers of HFD-fed Htr2a LKO mice. Lists of significantly less enriched GO gene sets in livers of HFD-fed Htr2a LKO mice analyzed by GSEA.

For both sheets, nominal P value (NOM P-val) threshold of 0.05 across all lists. Column "Name" indicates the name of GO gene sets. Column "NES" indicates normalized enrichment score, with gene sets enriched in Htr2a LKO mice having a positive NES, and gene sets less enriched in Htr2a LKO mice having a negative NES. Column "NOM P-val" indicates nominal P value. Column "FDR q-val" indicates false discovery rate q value.
